# High-frequency use of corrections, health, and social services, and association with mental illness and substance use

**DOI:** 10.1186/s12982-015-0040-9

**Published:** 2015-12-18

**Authors:** Julian M. Somers, Stefanie N. Rezansoff, Akm Moniruzzaman, Carmen Zabarauckas

**Affiliations:** Faculty of Health Sciences, Simon Fraser University, Burnaby, BC Canada; Department of Criminology, Simon Fraser University, Burnaby, BC Canada

## Abstract

**Background:**

A subgroup of individuals becomes entrenched in a “revolving door” involving corrections, health, and social welfare services. Little research has investigated the numbers of people that are in frequent contact with multiple public agencies, the costs associated with these encounters, or the characteristics of the people concerned. The present study used linked administrative data to examine offenders who were also very frequent users of health and social services. We investigated the magnitude and distribution of costs attributable to different categories of service for those in the top 10 % of sentences to either community or custodial settings. We hypothesized that the members of these subgroups would be significantly more likely to have substance use and other mental disorders than other members of the offender population.

**Methods:**

Data were linked across agencies responsible for services to the entire population of British Columbia spanning justice, health, and income assistance. Individuals were eligible for inclusion in the study if they were sentenced at least once in the Vancouver Provincial Court between 2003 and 2012. We examined the subset of participants who fell within the top 10 % of sentences and at least two of the following service categories: community physician services; hospital days; pharmaceutical costs; or income assistance between 2007 and 2012. We examined two groups of offenders separately (those in the top ten percent sentenced to community supervision or to custody) due to differences in time at risk and availability to receive community-based services.

**Results:**

From more than 14,000 offenders sentenced in Vancouver’s Downtown Eastside, very High Frequency service users associated with community (n = 216) and custody (n = 107) sentences incurred average attributable public service costs of $168,000 and $247,000 respectively over a 5-year period of observation. Health-related costs for both groups were over $80,000 per person, primarily associated with hospital admissions. Across both groups, 99 % had been diagnosed with at least one mental disorder and over 80 % had co-occurring substance use and another mental disorder.

**Conclusions:**

A subset of offenders with concurrent psychiatric disorders receives extremely high levels of service from health, social welfare, and justice sectors in close temporal succession. Members of this subpopulation require targeted supports in order to produce positive outcomes and prevent the perpetuation of a costly and ineffective revolving door.

**Electronic supplementary material:**

The online version of this article (doi:10.1186/s12982-015-0040-9) contains supplementary material, which is available to authorized users.

## Background

Criminal offenders have disproportionately high levels of health and social service needs when compared to the general population, including socioeconomic challenges related to employment and housing [1] and health-related needs involving mental illness and substance use [2, 3]. Inadequate responses to health and social needs are not only associated with initial involvement with the justice system, but contribute to the risk of criminal recidivism, creating a dynamic that many observers have characterized as a “revolving door” to the corrections system, particularly for people with addictions and mental illness (e.g., [4–6]). The gap between offender needs and the delivery of services is illustrated by US research indicating that while more than half of prison inmates are drug dependent [7], at most 10 % receive treatment while incarcerated [8]. Canadian research demonstrates that offenders with co-occurring substance use and mental disorders are at significantly higher risk of being reconvicted than other offenders, including those with mental disorders alone [2].

Several U.S. studies have examined the costs associated with chronic offenders (e.g., [9, 10]). However, economic estimates concerning the offender population have tended to focus on the costs of crime-related events [9], considering factors such as insurance, material and personal loss, and the administration of justice [11, 12]. Very few studies have examined the costs associated with individual offenders based on their involvement with public agencies and services. Researchers examining the histories of offenders with both substance use and mental disorders have reported: “few studies have been designed to investigate what types and amounts of treatment this population actually consumes” [13]. It is unclear how different forms of health service use influence the risk of recidivism, or the characteristics of those participants most likely to become involved with high rates of health *and* correctional services. Identifying the characteristics of offenders who are High Frequency service users may have implications for the promotion of health as well as public safety, establishing needs for targeted supports in order to promote recovery and prevent further recidivism.

The current study investigated the costs and characteristics of offenders who are high-frequency and concurrent users of healthcare, social services, and corrections. Offenders leaving custody have been identified as having distinct challenges regarding engagement with health and social services [14], and may therefore access lower levels of service than offenders sentenced directly to community supervision. People in custody also have fewer opportunities to engage community services concurrent with their sentences and must transition between jail and community settings. Offenders with the highest frequency of community sentencing experience substantial periods of supervision, including sanctions to reduce the risk of recidivism. It is therefore important to examine service volumes separately for those sentenced with the highest frequency to custody or community settings. We defined, a priori, high-frequency users as those who were in the top decile of health, social assistance, and correctional services. Flexible inclusion criteria for health and social services were adopted (i.e., two of: physician services; medication; hospitalization; social assistance) in order to identify offenders who were in very frequent contact with at least two distinct systems including corrections. The study had two objectives: to investigate the magnitude and distribution of costs attributable to different categories of service for those sentenced to community or custody; and to examine the role of mental disorders and substance use disorders among high-frequency users. We hypothesized that individuals in both high-frequency groups would have a higher prevalence of substance use disorders, mental disorders, and co-occurring disorders than other members of the offender population.

## Methods

### Ethical statement

This study involved the analysis of de-identified records and was approved by the Research Ethics Board of Simon Fraser University and the applicable research committees, privacy committees, and offices of the Assistant Deputy Ministers within the Government of British Columbia. This research was preceded by privacy impact assessments conducted by each contributing ministry and the host university, and subject to information sharing agreements between all participating institutions. Due to the de-identified nature of the data neither written nor verbal consent was possible.

### Data sources

We examined linked administrative data (2002–2012), spanning three provincial government ministries: Justice; Health Services; and Social Development and Social Innovation. Data from the contributing ministries comprise a relatively complete inventory of the health, justice, and income assistance services used by members of the British Columbia population. The completeness of these data reflects the central organizational and funding role provided by the provincial government in the administration of these various program areas. Matching of cases followed a multi-stage probabilistic algorithm and resulted in an 89 % match rate [2]. Citizens of British Columbia are legally required to register with the province’s publicly funded health system and are assigned a unique ID. Government departments can use this ID to link information from different program areas. All personal identifiers are deleted and replaced with an arbitrarily chosen study ID prior to export from government in order to create a de-identified research database.

Corrections-related incidents and sociodemographic variables (age, gender, ethnicity, education) were collected by the Ministry of Justice. The age of participants was treated as a continuous variable in the analysis. Due to the nature of administrative data, we are unable to specify if the gender variable represents self-reported gender—a social construct, or self-reported sex—a biological construct. We refer to this variable as gender, with this limitation noted. Ethnicity was limited to three groups: White, Aboriginal (Aboriginal, Métis, First Nations, Native or Inuit) and Other (Asian, Black, East Indian, Hispanic or other category). Education level was defined as highest level of formal education obtained and was analyzed by collapsing into four groups: Grade 9 or less, grade 10 or 11, grade 12 and vocational or university. Information regarding physician services delivered through the publicly supported Medical Services Plan (MSP), diagnoses, hospital separations, and publicly funded medications (PharmaCare) are maintained by the Ministry of Health. Financial support for shelter, disability, and forms of hardship is maintained by the Ministry of Social Development and Social Innovation. Unit costs for all services were provided directly by the ministry responsible.

### Participants

#### Provincial court sample

The sample was drawn from the full population of offenders in British Columbia (BC). Individuals were eligible for inclusion in the study if they were sentenced at least once in the Vancouver Provincial Court between April 1, 2003 and March 31, 2012. This 9 year period was used to establish eligibility based on correctional involvement. The sentencing court is located in Vancouver’s Downtown Eastside neighborhood, and receives cases from the surrounding geographic region. Prior population based research demonstrated that people with complex co-occurring psychiatric disorders and economic hardship were disproportionately concentrated in this geographical location compared to other regions of BC [15]. Exclusion criteria were as follows: age less than 18 at the time of enrolment (April 1, 2007); and not linkable to health data. Due to changes in data entry practices, details required to calculate the number of days served in custody and community supervision became fundamentally more comprehensive and reliable in 2007. We therefore used a 5 year time period (April 1, 2007 and March 31, 2012) to measure service use and other related characteristics.

#### High frequency service use samples

Two subsamples were created from the resulting group of eligible offenders: one group comprised of individuals who were in the top decile of days supervised on community orders; the second group consisting of those who were in the top decile of days served in custody.

Selection criteria for the two groups were as follows.

#### High frequency community supervision group

 Participants who were in the top decile of community supervision days were included if they were also in the top decile among recipients of at least two of the following service categories: community medical and lab service costs, days spent in hospital, publicly funded medication costs, or financial support payments all within the 5 year observation period.

#### High frequency custody group

Participants in the top decile of custody days were included if they were also in the top decile among recipients of at least two of the following service categories: community medical and lab service costs, days spent in hospital, publicly funded medication costs, or financial support payments all within the 5 year observation period.

### Comparison group participants (all *other* offenders)

Socio-demographic and corrections related variables were tabulated for the total offender population and for each of the two High Frequency service use groups. Statistical comparisons were made between each of the High Frequency service use groups and the remainder of the offender population (i.e., all other offenders meeting inclusion criteria but not in either respective top decile group).

### Mental disorders

MSP records (based on the ICD-9) were examined for diagnoses of mental disorders administered by physicians anytime between April 1, 2007 and March 31, 2012. This 5 year period was used to establish eligibility based on health service involvement among offenders convicted between 2003 and 2012 (see above). All disorders were included within the ICD range of 290–319 (mental disorders). Substance use disorders (SUD) were identified using the three-digit codes of 291, 292, 303, 304, and 305. Non-substance use mental disorders (NSMD) consisted of all other codes within the range identified. Details of these variables have been described elsewhere [2, 16].

### Statistical analysis

Continuous variables (e.g., age, service costs) were reported as means and standard deviations while categorical variables (e.g., gender, education level) were reported as proportions. Because we used the 90th percentile as a cut off, we also reported medians (50th percentile) and 90th percentiles for continuous variables. Between groups comparisons were conducted using between sample t tests (continuous variables) and Pearson’s Chi square or Fischer’s exact tests (nominal variables) where appropriate. If cell counts were less than five, test statistics based on Fischer’s exact test were reported.

Rates of service use per person–per year (PP/PY) were calculated using total number of services/cost as numerators and total person times at risk as denominators. Time at risk was calculated by subtracting the number of custody days from the maximum exposure time (5 years). Finally, Rate Ratios (RR) were estimated for each person and reported as effect sizes. Unadjusted Rate Ratios were estimated by dividing the rate for each identified High Frequency group by the rate for each comparison group.

Due to the over-dispersion of service use and other related variables, Negative binomial regression (NBR) analysis with Robust standard errors was used to obtain Adjusted Rate Ratios and corresponding 95 % Confidence intervals (CI). In the regression analyses, we used the log transformed (natural) time at risk as an offset variable in the model to adjust for variation in exposure period. In addition, socio-demographic variables (i.e., age at enrolment, gender, ethnicity, education level) were included in the NBR analysis to control for the effects of potential confounders. Adjusted models were used in order to derive unbiased estimates of Rate Ratios by controlling for the effects of confounders, not to support causal inferences. All reported p values were two sided. All costs were adjusted for inflation and converted to 2012 Canadian dollars (based on rates obtained on July 10, 2013 from the Bank of Canada. IBM SPSS Statistics 21.0 [17] and STATA 12 [18] were used to conduct these analyses.

## Results

### Characteristics of the two high frequency service use groups and those of all offenders

Between April 1, 2003 and March 31, 2012 a total of 14,372 individuals were sentenced at the Provincial Court in Vancouver and met our inclusion criteria (i.e., were at least 18 years of age and had linkable health data). Service use volumes and categories were examined over a 5-year period ending March 31, 2012. Throughout this period, the top 10 % of offenders sentenced to community supervision (n = 1443) served a minimum of 912 days, while the top 10 % of offenders sentenced to custody (n = 1442^1^) were incarcerated for a minimum of 325 days.

Two hundred and sixteen of the offenders in the top 10 % of community supervision days were also in the top decile of at least two categories of health and social service use and comprise the High Frequency (HF) Community Group. One hundred and seven of the offenders in the top 10 % of custody days satisfied the same additional service use criteria and comprise the HF Custody Group. Thirty-two individuals met criteria for both groups. Socio-demographic and justice-related characteristics for the total sample (All Offenders) are summarized in Table 1 alongside the corresponding results for each of the two High Frequency service-using samples. The value of the 90th percentile is provided for variables consisting of ratio data, including the variables used to define the subgroups of High Frequency service users. We calculated costs associated with correctional services consisting of days in custody and days under community supervision. Mean 5-year correctional costs were $21,085 for Provincial Court Offenders overall, $39,510 for those in HF Community and $123,466 for those in HF Custody.

**Table 1:** Socio-demographic and Justice related characteristics for all Provincial Court Offenders and High Frequency service using offenders (community and custody)

Variables	All offenders-Provincial Court (n = 14,372)	High Frequency Community Supervision (n = 216)	High Frequency Custody (n = 107)
	Mean (SD)/n (%)	Mean (SD)/n (%)	Mean (SD)/n (%)
Age at enrolment in years^a^			
Mean (SD)	35.7 (10.8)	38.5 (9.6)	37.3 (10.4)
Median and 90th percentile	35, 50	38, 51	37, 52
Female	2045 (14)	54 (25)	16 (15)
White	7904 (57)	146 (69)	74 (71)
Aboriginal	2092 (15)	36 (17)	19 (18)
Education—grade 9 or less	1717 (13)	27 (13)	17 (17)
Education—vocational/university	2492 (19)	40 (19)	12 (12)
Mental disorder diagnosis			
No mental disorder	5839 (41)	2 (1)	0 (0)
Only NSMD	2667 (19)	24 (11)	6 (6)
Only SUD	1517 (11)	12 (6)	7 (6)
Both	4349 (30)	178 (82)	94 (88)
# of sentences in past 5 years			
Mean (SD)	4.1 (6.7)	9.8 (8.3)	19.4 (11.0)
Median and 90th percentile	1, 12	7, 21	19, 34
# of jail sentences in past 5 years			
Mean (SD)	2.2 (4.6)	4.6 (5.8)	12.7 (7.9)
Median and 90th percentile	0, 8	3, 12	11, 24
# of any offences in past 5 years			
Mean (SD)	3.0 (5.1)	7.3 (6.4)	15.0 (9.0)
Median and 90th percentile	1, 9	6, 14	13, 28
# of property offences in past 5 years			
Mean (SD)	1.3 (3.1)	3.8 (4.9)	7.5 (7.0)
Median and 90th percentile	0, 4	2, 11	6, 18
# of breach offences in past 5 years			
Mean (SD)	0.8 (1.9)	1.8 (2.8)	4.5 (4.6)
Median and 90th percentile	0, 3	1, 5	3, 10
# of violent offences in past 5 years			
Mean (SD)	0.5 (1.2)	0.9 (1.2)	1.7 (2.7)
Median and 90th percentile	0, 2	0, 3	1, 5
# of community days in past 5 years			
Mean (SD)	347.3 (375.5)	1155.1 (194.5)	631.2 (516.6)
Median and 90th percentile	265, 912	1112, 1462	644, 1402
# of custody days in past 5 years			
Mean (SD)	93.2 (206.6)	158.4 (214.4)	590.9 (246.5)
Median and 90th percentile	0, 325	71, 416	511, 1021
Justice system costs in past 5 years ($CAD)^b^			
Mean (SD)	21,085 (42,510)	39,510 (43,702)	123,466 (49,813)
Median and 90th percentile	2958, 69, 690	21, 407, 92,778	107, 862, 210, 386

*SD* Standard deviation

^a^Age was calculated at April 1, 2007

^b^Includes cost of Community Supervision ($6.5 per day) and cost of custody ($202 per day)

Table 2 presents health and social service details for the three groups including mean costs associated with categories of service over the previous 5 years. Physician service costs were $3,809 (All Offenders), $14,477 (HF Community) and $15,493 (HF Custody). Medication costs were $3,809 (All Offenders), $15,950 (HF Community) and $11,338 (HF Custody). Social Assistance payments were $16,757 (All Offenders), $46,962 (HF Community), and $38,088 (HF Custody). The mean number of days spent in hospital was 8.5 for All Offenders, and 51.5 and 58.5 for the HF Community and HF Custody groups respectively. Per person costs for health, corrections, and social assistance were $53,003 for All Offenders, $168,389 for those in the HF Community group, and $246,899 for those in the HF Custody group over the 5 years observed.

**Table 2:** Health and Social Services for Provincial Court participants and High Frequency groups during the study period

Variables	All offenders-Provincial Court (n = 14,372)	High Frequency Community Supervision (n = 216)	High Frequency Custody (n = 107)
	Mean (SD)	Mean (SD)	Mean (SD)
# of MSP services in past 5 years			
Mean (SD)	132.6 (195.1)	432.4 (240.3)	432.9 (292.3)
Median and 90th percentile	55, 393	405, 758	405, 743
MSP costs in past 5 years ($CAD)			
Mean (SD)	3809 (5610)	14, 477 (8470)	15, 493 (8482)
Median and 90th percentile	1699, 10, 284	12, 219, 24, 970	12, 808, 28, 522
# of acute hospital admissions in past 5 years			
Mean (SD)	1.0 (2.6)	5.0 (6.0)	6.9 (7.9)
Median and 90th percentile	0, 3	3, 12	4, 15
Hospital days in past 5 years ($CAD)			
Mean (SD)	8.5 (31.7)	51.5 (79.7)	58.5 (62.6)
Median and 90th percentile	0, 19	24, 134	38, 154
Prescription costs in past 5 years ($ CAD)			
Mean (SD)	3809 (5610)	15, 950 (13,577)	11, 338 (8974)
Median and 90th percentile	1699, 9166	13, 602, 31,905	9838, 23,765
Health care costs in past 5 years ($CAD)^a^			
Mean (SD)	15,160 (38, 704)	81,918 (84,698)	85,344 (68,767)
Median and 90th percentile	2428, 38,638	54,071, 167,311	59,610, 196,400
# of social assistance payments in past 5 years			
Mean (SD)	21.6 (24.1)	52.9 (14.3)	47.9 (17.0)
Median and 90th percentile	10, 60	58, 60	51, 60
Social assistance payments in past 5 years ($CAD)			
Mean (SD)	16,757 (21,698)	46,962 (18,370)	38,088 (17,967)
Median and 90th percentile	5180, 53,277	50,788, 65,278	37,700, 62,290
Total costs in past 5 years ($CAD)^b^			
Mean (SD)	53,003 (66,741)	168,389 (91,392)	246,899 (75,667)
Median and 90th percentile	28,861, 136,340	140,007, 289,311	229,381, 360,271

*MSP* Medical services plan

^a^Includes costs for MSP services, hospital days ($1000 per day) and prescription medications

^b^Includes costs for corrections-related services (Community Supervision and custody days), health care (MSP services, hospital days and prescriptions) and social assistance

### Statistical comparisons of offender characteristics

Both HF subgroups (community and custody) were compared to all other offenders on socio-demographic and diagnostic variables (Additional file 1). Members of the HF Community subgroup were significantly older (mean 38.5 vs 35.5 years), significantly more likely to be female (25 vs 14 %), and significantly more likely to be White (69 vs 57 %) than other offenders. There were no significant differences between the groups based on educational achievement. Psychiatric status was determined based on physician diagnoses administered anywhere in British Columbia during the 5 year observation period. Of those not included in the HF Community group (n = 14,156), 41 % had no diagnosis, 19 % had a mental disorder diagnosis, 11 % had a substance-related diagnosis, and the remaining 29 % had both a substance related and a non-substance related mental disorder diagnosis. In contrast, 1 % of the HF Community group had no psychiatric diagnosis and 82 % had been diagnosed with concurrent substance and mental disorders.

The HF Custody group did not differ significantly from other offenders based on either average age or gender. However, they were significantly more likely to be White and had a lower level of educational achievement than the remainder of the offender population. Every member of the HF Custody group had at least one psychiatric diagnosis and 88 % were diagnosed with both a substance-related and a non-substance related mental disorder.

### Relative differences between groups in rates of services and events

Relative differences in health, social, and corrections events between the two HF cohorts (Community and Custody) compared with all non-HF offenders were estimated using Rate Ratios (see Tables 3 and 4). The rate of occurrence of each event (or service) was calculated per-person per-year, after adjusting for time at risk due to hospitalization. Unadjusted Rate Ratios (URR) indicate the degree of difference between groups. All of the planned comparisons between the HF Community group and their “non-HF” counterparts were significant.

**Table 3:** Rate ratio estimates of High Frequency Community Supervision compared to all other offenders

Variables	High Frequency Community Supervision (n = 216), Rate PP/PY	All Other Offenders (n = 14,156), Rate PP/PY	Unadjusted Rate Ratio^a^	Adjusted Rate Ratio (95 % CI)^b^
Any offence	1.6	0.6	2.5	2.9 (2.3, 3.6)
Property offence	0.8	0.3	3.2	3.6 (2.6, 4.9)
Breach offence	0.4	0.2	2.5	3.5 (2.4, 5.0)
Violent offence	0.2	0.1	2.0	2.2 (1.8, 2.8)
Community supervision days^d^	253.0	70.6	3.6	3.6 (3.4, 3.9)
Days in custody^c^	31.7	18.4	1.7	2.9 (1.9, 4.3)
MSP services	94.7	27.0	3.5	3.2 (2.9, 3.5)
MSP costs^d^ ($CAD)	3170.3	768.0	4.1	3.9 (3.5, 4.3)
Acute hospital admissions	1.1	0.2	5.7	5.7 (4.6, 6.9)
Hospital days^d^	11.3	1.7	6.8	6.6 (5.2, 8.3)
Prescription costs^d^ ($CAD)	3493.1	552.3	6.3	6.4 (5.5, 7.5)
# of social assistance payments	11.6	4.5	2.6	2.5 (2.3, 2.8)
Social assistance payments ($CAD)	10284.5	3432.5	3.0	3.0 (2.6, 3.4)

*MSP* Medical services plan

^a^Rate was calculated based on time at risk (exposure time-custody day). Unadjusted Rate Ratio was calculated from the rate for HF Community by dividing the rate of the non-HF Community

^b^Negative binomial regression analysis was conducted to obtain Adjusted Rate Ratio after controlling the effects of time at risk and socio-demographics (age, gender ethnicity and education level). Confidence intervals were estimated using Robust Standard Errors

^c^Calculation was based on the entire exposure time, not by time at risk

^d^Variables used to determine community High Frequency user groups

**Table 4:** Rate ratio estimates of High Frequency Custody compared to all other offenders

Variables	High Frequency Custody (n = 107), Rate PP/PY	All Other Offenders (n = 14,265), Rate PP/PY	Unadjusted Rate Ratio^a^	Adjusted Rate Ratio (95 % CI)^b^
Any offence	4.4	0.6	7.2	7.4 (6.0, 9.0)
Property offence	2.2	0.3	8.5	8.8 (6.5, 11.8)
Breach offence	1.3	0.2	8.6	10.0 (7.3, 13.8)
Violent offence	0.5	0.1	5.1	5.6 (3.9, 8.1)
Community supervision days	186.6	72.6	2.6	2.6 (2.2, 3.1)
Days in custody^c,d^	118.2	17.9	6.6	8.8 (6.5, 11.9)
MSP services	128.0	27.4	5.9	4.6 (4.0, 5.3)
MSP costs^c^ ($CAD)	4580.8	782.6	4.2	6.0 (5.3, 6.9)
Acute hospital admissions	2.0	0.2	10.5	11.2 (8.6, 14.7)
Hospital days^c^	17.3	1.7	10.1	10.7 (8.4, 13.6)
Prescription costs^c^ ($CAD)	3352.2	580.1	5.8	6.5 (5.2, 8.0)
# of social assistance payments	14.2	4.5	3.1	3.1 (2.7, 3.7)
Social assistance payments ($CAD)	11261.4	3940.4	3.2	3.4 (2.8, 4.1)

*MSP* Medical services plan

^a^Rate was calculated based on time at risk (exposure time-custody day). Unadjusted Rate Ratio was calculated from the rate for HF Custody by dividing the rate of the non-HF Custody

^b^Negative binomial regression analysis was conducted to obtain Adjusted Rate Ratio after controlling the effects of time at risk and socio-demographics (age, gender ethnicity and education level). Confidence Intervals were estimated using Robust Standard Errors

^c^Variables used to determine custody High Frequency user groups

^d^Calculation was based on exposure time, not by time at risk

Adjusted Rate Ratios (ARR) are listed, controlling for time at risk as well as age, gender, ethnicity, and educational achievement. Rates for all categories of service use—not merely those used to define High Frequency groups—were significantly higher for both HF groups. When compared to all other offenders, the HF Community group incurred 6.6 times the number of hospital days, prescription drug costs that were 6.3 times larger and physician costs that were 3.9 times larger (Table 3). Members of the HF Community cohort had 3.6 times the number of community supervision days as the remainder of the offender population, reflecting the fact that this was the base selection criterion used to define the cohort. A smaller but nonetheless significant difference was observed for the amount received in social assistance payments (3.0 times).

Comparisons between the HF Custody group and all other offenders are presented in Table 4. Results show that the HF Custody group incurred 10.7 times the number of hospital days, prescription drug costs that were 6.5 times larger and physician costs that were 6.0 times larger than those of other offenders. Reflecting our selection criteria, the two groups differed in the number of days served in custody (8.8 times). Again, a smaller but nonetheless significant difference was observed for the amount received in social assistance payments (3.4 times).

## Discussion

To our knowledge, this is the first Canadian study to investigate the costs and characteristics of offenders who are high-frequency users of healthcare, social services, and corrections concurrently. We separately examined two cohorts comprised of those who served the highest number of days in custody (n = 107), and those who served the highest number of days under community supervision (n = 216). Of more than 14,000 offenders sentenced in Vancouver’s Downtown Eastside, those included in each cohort were also in the top decile for more than one category of health and social services use. Consistent with our hypothesis, 99 % of the members of both cohorts had been diagnosed with a psychiatric disorder, and over 80 % had been diagnosed with *both* a substance use disorder and another mental disorder.

Per person costs over 5 years were $168,389 in the High Frequency Community group, with nearly half of this total attributed to health-related services ($81,918). The largest single contributor to the total cost for this cohort was stays in hospital ($51,500). Additional health-related costs were comprised of physician payments ($14,477) and publicly funded medication costs ($15,950). Social assistance payments averaged $46,962 per person over the same five-year period with payments made in 53 out of 60 months.

Individuals in the High Frequency Custody group incurred average costs of $246,899 over 5 years. Corrections-related costs accounted for half of this amount ($123,466). Individuals in this group spent an average of 591 days in custody during the 5-year observation period. Notably, despite the amount of time they spent in custody, the members of this cohort had considerable involvement with community-based health and social services. In fact, costs associated with health ($85,344) and social assistance ($38,088) were comparable to the amounts observed in the High Frequency Community cohort. When summed, the cost of services provided over 5 years to both groups was $26.5 million. These total amounts do not include other justice costs such as police, crown counsel, defence, or courts, nor do they include health services provided while in custody; many of which are administered and recorded separately from the public healthcare system used in our analyses.

The most striking difference between both of the High Frequency groups and other offenders was the prevalence of psychiatric diagnoses. While 41 % of the general offender population had no evidence of a psychiatric diagnosis through the province’s public healthcare system, only 1 % of the High Frequency community cohort and 0 % of the High Frequency Custody group had not been diagnosed in the 5-year observation period. More tellingly, the overwhelming majority in both of the High Frequency groups (82 and 88 %) had been diagnosed with a substance related as well as a non-substance related mental disorder. Co-occurring mental and substance use disorders have previously been shown to be a strong predictor of recidivism to the correction system [2, 3, 19]. The present study extends this finding by showing that offenders with co-occurring disorders were also associated with very high levels of service involvement across health and social welfare during the same 5-year span in which they were involved with corrections. The High Frequency of social assistance payments to participants attests to the chronicity of financial hardship within the study cohorts.

Our results demonstrate that involvement with relatively high volumes of public services was not sufficient to prevent recurrent correctional involvement among a subset of offenders. Further research is urgently needed to improve the health and public safety outcomes associated with these cohorts. The current findings quantify the partial costs of the status quo, providing a basis for planning and budgeting alternative interventions. Our findings also demonstrate that co-occurring mental disorders and substance use disorders are overwhelmingly prevalent among those who are involved with the highest rates of services across sectors, and suggest that improved interventions should be responsive to the needs of this subgroup.

The characteristics of High Frequency service-use offenders in our study are consistent with the results of other research (e.g., [20]). In addition, Vancouver police recently declared a public health crisis related to inadequately treated mental illness and substance use, and estimated that approximately 300 individuals in the downtown core of the city (from which the current cohort was drawn) have particularly acute needs that result in recurrent breaches of public safety [21]. These impressions, derived from the daily experiences of police officers over several years, match the results of the present study.

Recent research by our team found that the provincial prevalence of people with “complex co-occurring disorders” (defined on the basis of concurrent mental illness, substance dependence, multiple convictions, hospitalizations, and shelter assistance) varied widely between communities, but was high in rural and remote settings as well as urban ones [15]. The highest absolute number of people who met criteria for “complex co-occurring disorders” was in the Downtown Eastside, the same neighbourhood where the present study was mounted. These findings suggest that the dynamic of frequent criminal recidivism, other public service use, and concurrent mental disorders may not be restricted to urban areas where they are most visible. Further research is needed to establish the generalizability of the present study to other regions and to settings with different systems of social programs, health insurance, and justice practices than those in place in Downtown Vancouver.

Several limitations of this study should be acknowledged. Our reliance on administrative data may mask sources of bias such as coding errors, incompleteness, or inaccuracies associated with professional judgment. For example, the diagnosed prevalence of substance use and mental disorders among offenders is likely to reflect both false positive and false negative errors. These data also omit important forms of service, including emergency department visits, assessment and treatment by private psychologists and psychiatrists, as well as outreach and first responder services including police. Strengths of this study are the inclusion of multiple domains of relevant services, verified public costs, and multiple years of observation.

Considering the costs of correctional supervision and the prevalence of mental health-related needs among offenders, a number of solutions have been proposed to promote offender recovery and rehabilitation in the community. An area of dramatic growth has been the proliferation of specialized courts involving partnerships between health and community services [22]. Problem-solving courts that follow structured models and incorporate appropriate treatments are among the relevant models that have been found to be effective (e.g., [16, 23, 24]). Recent studies have also demonstrated the effectiveness of community-based services for offenders with severe and complex needs, including Forensic Assertive Community Treatment [25], Housing First [26], and Critical Time Interventions [27]. These inter-agency interventions go beyond a focus on symptom reduction and address core determinants of health and public safety (e.g., housing, employment, etc.) among individuals with complex substance use and mental disorders.

In the current study, offenders in both High Frequency cohorts spent seven times as many days in hospital as other offenders, and it is possible that they were in need of additional acute care. When compared to other offenders, the High Frequency custody group had seven times the number of hospital admissions, six times the number of property offence convictions, and six times the number of breach offence convictions. Our findings are indicative of lives spent in perpetual crisis. There is an urgent need to implement community-based services to reduce criminal recidivism and prevent recurrent medical crises by addressing the social determinants of these outcomes. Appropriately implemented, community-based initiatives hold the promise of producing superior outcomes for individual offenders as well as for the general public. In light of the significant costs associated with the status quo, further research is needed to investigate whether community-based diversionary initiatives may result in favourable cost avoidance for individuals such as those represented in this study.

## Additional file

10.1186/s12982-015-0040-9 Appendices A: Comparison of socio-demographic and diagnostic characteristics between HF Community and all other offenders and B: Comparison of socio-demographic and diagnostic characteristics between HF Custody and all other offenders.

## Footnotes

1 Due to rounding.
