# Supplementary material for: High-frequency use of corrections, health, and social services, and association with mental illness and substance use
Source: Emerg Themes Epidemiol. 2015 Dec 18;12:17. doi: 10.1186/s12982-015-0040-9 (PMC4683696; doi:10.1186/s12982-015-0040-9)
Supplement: Supplementary file 1 — 10.1186/s12982-015-0040-9 Appendices A: Comparison of socio-demographic and diagnostic characteristics between HF Community and all other offenders and B: Comparison of socio-demographic and diagnostic characteristics between HF Custody and all other offenders. [file 12982_2015_40_MOESM1_ESM.docx]

**Appendix A: Comparisons of socio-demographic and diagnostic characteristics between HF Community and all other offenders**

| **Variables** | **HF Community=no (n=14156)**  **Mean (SD) /N (%)** | **HF Community=yes (n=216)**  **Mean (SD) /N (%)** | P value^[[1]](#footnote-1)^ |
| --- | --- | --- | --- |
| Age at enrolment in years^[[2]](#footnote-2)^  Mean (SD) | 35.7 (10.8) | 38.5 (9.6) | **<0.001** |
| Gender  Male  Female | 12162 (86)  1991 (14) | 162 (75)  54 (25) | **<0.001** |
| Ethnicity  White  Aboriginal  Other | 7758 (57)  2056 (15)  3773 (28) | 146 (69)  36 (17)  30 (14) | **<0.001** |
| Education level  Grade 9 or less  Grade 10/11  Grade 12  Vocational /University | 1690 (13)  3928 (31)  4678 (37)  2452 (19) | 27 (13)  74 (35)  69 (33)  40 (19) | 0.538 |
| History of mental disorder in past five years  No mental disorder  Only NSMD  Only SUD  Both | 5837 (41)  2643 (19)  1505 (11)  4171 (29) | 2 (1)  24 (11)  12 (6)  178 (82) | **<0.001** |

**Appendix B: Comparisons of socio-demographic and diagnostic characteristics between HF Custody and all other offenders**

| **Variables** | **HFU-no (n=14265)**  **Mean (SD) /N (%)** | **HFU-yes (n=107)**  **Mean (SD) /N (%)** | P value^[[3]](#footnote-3)^ |
| --- | --- | --- | --- |
| Age at enrolment in years^[[4]](#footnote-4)^  Mean (SD) | 35.7 (10.8) | 37.3 (10.4) | 0.124 |
| Gender  Male  Female | 12233 (86)  2029 (14) | 91 (85)  16 (15) | 0.830 |
| Ethnicity  White  Aboriginal  Other | 7830 (57)  2073 (15)  3792 (28) | 74 (71)  19 (18)  11 (10) | **0.001** |
| Education level  Grade 9 or less  Grade 10/11  Grade 12  Vocational /University | 1700 (13)  3961 (31)  4716 (37)  2480 (19) | 17 (17)  41 (40)  31 (31)  12 (12) | **0.047** |
| History of mental disorder in past five years  No mental disorder  Only NSMD  Only SUD  Both | 5839 (41)  2661 (19)  1510 (10)  4255 (30) | 0 (0)  6 (6)  7 (6)  94 (88) | **<0.001** |

1. -Independent sample t test and non-parametric test (Pearson’s chi-squared or Fisher’s Exact test as appropriate) were conducted to compare continuous and categorical variables between groups respectively [↑](#footnote-ref-1)
2. -Age was calculated at April 1^st^ of 2007 [↑](#footnote-ref-2)
3. - Independent sample t test and non-parametric test (Pearson’s chi-squared or Fisher’s Exact test as appropriate) were conducted to compare continuous and categorical variables between groups respectively [↑](#footnote-ref-3)
4. -Age was calculated at April 1^st^ of 2007 [↑](#footnote-ref-4)
